# Peroxynitrite Generation and Increased Heterotrophic Capacity Are Linked to the Disruption of the Coral–Dinoflagellate Symbiosis in a Scleractinian and Hydrocoral Species

**DOI:** 10.3390/microorganisms7100426

**Published:** 2019-10-09

**Authors:** Laura Fernandes de Barros Marangoni, Miguel Mies, Arthur Z. Güth, Thomás N. S. Banha, Alex Inague, Juliana da Silva Fonseca, Camila Dalmolin, Samuel Coelho Faria, Christine Ferrier-Pagès, Adalto Bianchini

**Affiliations:** 1 Pós-Graduação em Oceanografia Biológica, Oceanographic Institute, Federal University of Rio Grande, Av. Itália, Km 8, Rio Grande, RS 96203-900, Brazil; adaltobianchini@furg.br; 2 Coral Vivo Institute, Rua dos Coqueiros, Parque Yaya, Santa Cruz Cabrália, BA 45.807-000, Brazil; miguel.mies@usp.br; 3 Centre Scientifique de Monaco, Marine Department, 8 Quai Antoine 1er, MC-98000, Monaco; ferrier@centrescientifique.mc; 4 Oceanographic Institute, University of São Paulo. Praça do Oceanográfico, 191-05508-120 São Paulo, SP, Brazil; azguth@gmail.com (A.Z.G.); sotobanha@gmail.com (T.N.S.B.); 5 Chemistry Institute, University of São Paulo. Av Prof Lineu Prestes, 748-05508-000 São Paulo, SP, Brazil; inague.alex@gmail.com; 6 Pós-Graduação em Ciências Fisiológicas, Biological Science Institute, Federal University of Rio Grande, Av. Itália, Km 8, Rio Grande, RS 96203-900, Brazilcamidal@gmail.com (C.D.); 7 Institute of Biosciences, University of São Paulo. São Paulo, SP 05508-090, Brazil; scoelhofaria@gmail.com; 8 Bermuda Institute of Ocean Sciences, St. George’s GE01, Bermuda

**Keywords:** coral reefs, heterotrophy, hydrocorals, oxidative stress, reactive nitrogen species

## Abstract

Ocean warming is one of the greatest global threats to coral reef ecosystems; it leads to the disruption of the coral–dinoflagellate symbiosis (bleaching) and to nutrient starvation, because corals mostly rely on autotrophy (i.e., the supply of photosynthates from the dinoflagellate symbionts) for their energy requirements. Although coral bleaching has been well studied, the early warning signs of bleaching, as well as the capacity of corals to shift from autotrophy to heterotrophy, are still under investigation. In this study, we evaluated the bleaching occurrence of the scleractinian coral *Mussismillia harttii* and the hydrocoral *Millepora alcicornis* during a natural thermal stress event, under the 2015–2016 El Niño influence in three reef sites of the South Atlantic. We focused on the link between peroxynitrite (ONOO^−^) generation and coral bleaching, as ONOO^−^ has been very poorly investigated in corals and never during a natural bleaching event. We also investigated the natural trophic plasticity of the two corals through the use of new lipid biomarkers. The results obtained first demonstrate that ONOO^−^ is linked to the onset and intensity of bleaching in both scleractinian corals and hydrocorals. Indeed, ONOO^−^ concentrations were correlated with bleaching intensity, with the highest levels preceding the highest bleaching intensity. The time lag between bleaching and ONOO^−^ peak was, however, species-specific. In addition, we observed that elevated temperatures forced heterotrophy in scleractinian corals, as *Mu. harttii* presented high heterotrophic activity 15 to 30 days prior bleaching occurrence. On the contrary, a lower heterotrophic activity was monitored for the hydrocoral *Mi. alicornis*, which also experienced higher bleaching levels compared to *Mu. hartii*. Overall, we showed that the levels of ONOO^−^ in coral tissue, combined to the heterotrophic capacity, are two good proxies explaining the intensity of coral bleaching.

## 1. Introduction

Ocean warming is one of the greatest global threats to coral reef ecosystems [1]. Such a threat can be even more intense during pulse heat stress events, such as those occurring during the El Niño Southern Oscillation (ENSO) [2]. The 2015–2016 ENSO, which caused unprecedented elevation in seawater temperatures, triggered the third global-scale event of mass coral bleaching [2,3], affecting 75% of the world’s reefs [1]. Coral bleaching is a complex physiological process characterized by the disruption of the symbiosis between the coral host and its endosymbiotic Symbiodiniaceae dinoflagellates [4,5]. Since dinoflagellates provide most of the nutritional needs of the symbiotic association through their autotrophic acquisition of energy [6,7], their loss can severely impact the corals’ energetic state, and on the long term, can lead to coral death. Many studies have thus focused on understanding the factors and processes that trigger coral bleaching. 

At the physiological level, it has been demonstrated that coral species able to shift from an autotrophic to a heterotrophic mode of nutrition (i.e., plankton predation by the coral host) can be more resistant or resilient to bleaching [8,9]. Although the natural heterotrophic capacity of corals is difficult to assess, a recent study has developed fatty acid markers, which can quantitatively determine the predominance of autotrophy and heterotrophy in corals [10]. Specifically, stearidonic acid (SDA, 18:4ω3) and docosapentaenoic acid (DPA, 22:5ω3) are fatty acids that have been validated as trophic markers for autotrophic feeding in corals, while cis-gondoic acid (CGA, 20:1ω9) has been validated as a marker for heterotrophic feeding [10]. Temporal variations in these fatty acids are used to calculate the Predominant Trophic Mode Index (PTMI), which determines the predominant feeding mode between autotrophy and heterotrophy [10]. Such an index has, however, never been used to assess the trophic mode of corals experiencing a global thermal stress event.

At the cellular level, over two decades of research have implicated oxidants, including reactive oxygen and nitrogen species (ROS and RNS, respectively), as pivotal factors responsible for coral bleaching [11,12,13,14,15,16,17,18,19]. More specifically, under thermal stress conditions, increasing amounts of superoxide anion (O_2_^−^) can be produced in the inner mitochondrial membrane and in the chloroplasts [20]. Also, symbiotic cnidarians under thermal stress can present increased nitric oxide (NO) concentrations, a RNS implicated in numerous cell signaling and pathophysiological processes in eukaryotic cells [14]. In presence of O_2_^-^, NO can be converted into peroxynitrite (ONOO^−^), a very reactive RNS known to cause irreversible damages to biomolecules [21]. Although the implication of NO in coral bleaching has been previously studied in laboratory experiments [14,15,18,22,23], its role in the production of ONOO^−^, during natural bleaching events remains unclear [16]. 

In this field work, we specifically studied the bleaching occurrence of two coral species, the scleractinian *Mussismillia harttii* and the hydrocoral *Millepora alcicornis,* during a natural bleaching event occurring during the 2015–2016 El Niño. To further our knowledge on the role of heterotrophy and ONOO^−^ generation in bleaching susceptibility, we followed these two parameters, together with seawater temperatures, in three different reef sites located in the South Atlantic. This is the first attempt to link ONOO^−^ production and in situ coral bleaching, as well as to assess in situ heterotrophy of a scleractinian and hydrocoral using the PTMI index. 

## 2. Materials and Methods

### 2.1. Reef Sites

**Figure 1:**
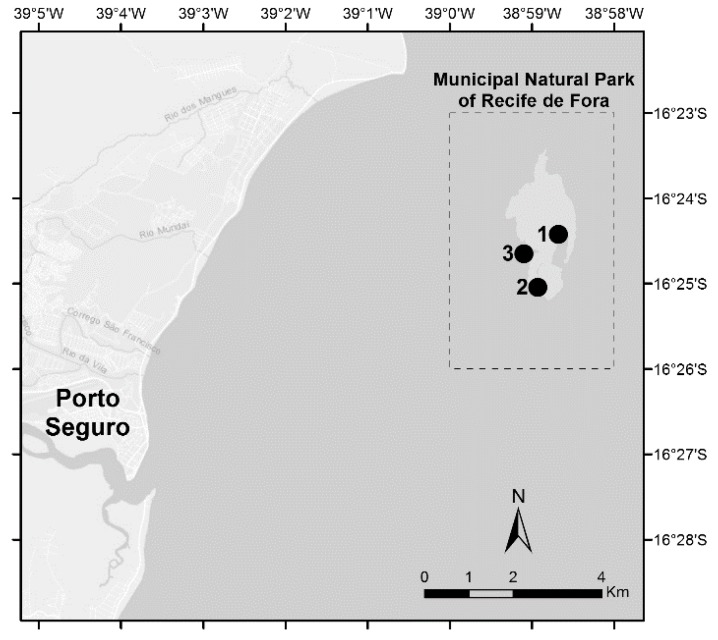
Study area of the Recife de Fora Marine Protected Area in the Southwestern Atlantic, Brazil. Reef sites: (1) Taquaruçu, (2) Mourão, (3) Funil. (Adapted from Marangoni et al. 2019).

Three reef sites located at the conservation area of the Municipal Natural Park of Recife de Fora (Porto Seguro, Bahia, Northeastern Brazil) were surveyed: (1) Taquaruçu (16° 24′ 25.3”S, 38° 58′ 41.0”W); (2) Mourão (16° 24′ 39.0”S, 38° 59′ 06.1”W); and (3) Funil (16° 25′02.6”S, 38° 58′ 55.8”W) (Figure 1). Repeated surveys of a particular location are typically less spatially comprehensive than stratified random surveys, however, are considered a useful sampling design to provide information on stress and degradation of coral reefs [24]. Therefore, the repeated surveys in representative sections of the Recife de Fora Marine Protected Area was considered the best approach, considering the temporal scale of our study and the aim to assess physiological responses in corals. The sites were selected according to the National Oceanic and Atmospheric Administration (NOAA) predictions for coral bleaching occurrence (https://coralreefwatch.noaa.gov/satellite/analyses_guidance/global_coral_bleaching_2014_17_status.php). In addition, they are included in the Brazilian Reef Check monitoring program (www.reefcheck.org, www.recifescosteiros.org.br), and are part of a recent Brazilian Conservation Plan for Endangered Species of Reef Environments (PAN Corais, ICMBio, 2016). *Mussismilia harttii* and *Mi. alcicornis* are the most abundant corals in these sites (unpublished data, E. N. Calderon) and were chosen in this study.

### 2.2. Thermal Stress Assessment

Local temperature conditions were assessed using in situ temperature data loggers (HOBO Pendant model UA-002-64) and historical data records obtained from NOAA′s 5 km virtual station, Recife de Fora, Brazil. In situ water temperature was continuously recorded every 15 min throughout the monitoring period (15, December, 2015 to 15, June, 2016). Data processing followed methods developed by NOAA Coral Reef Watch (CRW) program and ReefTemp monitoring system [25]. Data obtained from the NOAA virtual station for March 2013 to December 2015 were combined with in situ temperature data from December 2015 onwards, to calculate: (i) the onset of potential coral bleaching, which was defined to begin at degree heating weeks (DHW) values of 4 °C-weeks or greater; and (ii) sea surface temperature anomaly (SSTA), calculated as the positive deviations of daily average temperatures from the long-term (4 years) average temperature observed for each month. DHW are the accumulation of temperature anomalies exceeding the maximum of the monthly mean SST for a given region over the last 12-week period [26,27,28]. To calculate DHW, only anomalies ≥1 °C were considered [29].

### 2.3. Biological Survey

Colonies of the scleractinian coral *Mu. harttii* and the hydrocoral *Mi. alcicornis* were surveyed by SCUBA diving for changes in colony coloration every 15 days, from December 15th, 2015 to June 15th, 2016. One transect of 20 × 2 m, at a depth of 2 m (low tide), was haphazardly fixed each time and performed in each of the three sites. Colonies were photographed next to a PVC Coral Health Chart [30] along the fixed transects. The number of *Mu. harttii* and *Mi. alcicornis* colonies photographed in each of the transects varied from 8–34 and 18–33, respectively. Bleaching occurrence was evaluated following the modified coral watch protocol (www.reefquest.org). Colonies that were inside (at least in part) of each transect (20 × 2 m) were recorded, and coral colonies were considered “bleached” if at least 50% of its surface area was bleached. In addition, biological samples from both species were collected every 15 days for each site alongside all transects within an area of 0.04 km^2^. For each transect, 4 colonies per species were sampled, with one polyp for *Mu. harttii* and an apical piece of approximately 3 cm for *Mi. alcicornis*. Completely bleached colonies were avoided since they tend to loose tissue biomass (e.g., protein total content), which could affect biochemical assays conducted in the present study. Fragments were cut and immediately frozen in liquid nitrogen for subsequent analyses. Collections were performed under the permission of the Brazilian Environmental Agency (SISBIO).

### 2.4. Physiological Measurements

#### 2.4.1. Fatty Acids Extraction and Analysis

*Mussimilia harttii* and *Mi. alcicornis* fragments were weighed and lipid extraction was performed according to Mies et al. [31]. Coral tissues were crushed [32] and incubated with 50 µL of tridecanoic acid (C13:0, 1.0 mg mL^−1^), 1.85 mL of methanol, and 100 µL of acetyl chloride for 1 h at 100 °C. Hexane was added, the sample vortexed, and the upper organic phase transferred to a new vial. Evaporation was performed under a N flow_2_ until drying and the content was then resuspended in 100 µL of hexane. Fatty acid methyl esters (FAME) were analyzed on a Trace 1310 gas chromatograph (Thermo Scientific) equipped with a flame ionization detector. FAME were separated with a DB-FFAP column of 15 m × 0.1 mm ID x 0.1 µm film thickness (J & W Scientific, Agilent Technologies). The program started at 150 °C for 15 s, increasing 35 °C per minute up to 200 °C, and 8 °C per minute up to 250 °C and maintained for 4 min. Next, 1 µL of the sample was injected and carried by a mixture of hydrogen and nitrogen. 

The identification of stearidonic acid (SDA), docosapentaenoic acid (DPA), and cis-gondoic acid (CGA) was done by direct comparison of their retention times with 47033 PUFA Nº1 Marine Source (Sigma-Aldrich) standard mix. For each chromatogram, peaks were integrated and normalized by the internal standard. After the extraction, the skeleton fragments were washed with 2% NaClO for 2 min and dried at 63 °C for 30 h to remove organic tissue. The fragments were reweighed and both soft tissue and skeleton weight were determined. 

#### 2.4.2. Predominant Trophic Mode Index 

To determine which feeding mode prevailed, the data for SDA, DPA, and CGA concentration were computed into the equation below, calculating the Predominant Trophic Mode Index (PTMI). The PTMI is calculated based on the ratio between the temporal variations in the content of fatty acids related to both autotrophic (SDA and DPA) and heterotrophic (CGA) feeding [10]:

(1) $$\;\mathrm{PTMI}=\left[\left(\frac{\mathrm{autFATM}\left(t_{2}\right)}{\mathrm{autFATM}\left(t_{1}\right)}\right)-1]-[\left(\frac{\mathrm{hetFATM}\left(t_{2}\right)}{\mathrm{hetFATM}\left(t_{1}\right)}\right)-1\right]$$

where autFATM = µg of (SDA + DPA) g^−1^ of coral soft tissue, hetFATM = µg of CGA g^−1^ of coral soft tissue, t_1_ = initial sampling period, t_2_ = next sampling period. The result is dimensionless and indicates the prevalence of autotrophy in the case of positive values and the prevalence of heterotrophy for negative values. 

#### 2.4.3. Peroxynitrite (ONOO^−^) Quantification

The tissue of coral and hydrocoral samples was analyzed for ONOO^-^ concentration using the Amplitude^TM^ Fluorimetric Peroxynitrite Quantification Kit (AAT Bioquest^®^, Sunnyvale, CA, USA). The reagent DAX-J2^TM^ PON Green 99, specifically reacts with ONOO^−^, with high selectivity over ROS and other RNS, to generate a green fluorescent product detected at Ex/Em = 490/530 nm. Briefly, approximately 0.5 cm^2^ of each coral/hydrocoral sample was cut using a cutter (HD Stainless Steel Stony Coral Cutter, Ocean Wonders, USA) and homogenized in assay buffer solution (provided by the manufacturer) using a sonicator (Frequency 70 KHz, Sonaer Ultrasonics, Farmingdale, NY, USA). The remaining piece of skeleton was discarded and homogenates were centrifuged at 10,000 *g* (20 min, 4 °C). The intermediary phase was used to perform the assay, according to the manufacturer instructions. Coral tissue can present green autofluorescence that can increase under temperature stress depending on symbionts clade [33]. To ensure no interferences with our measurement, we performed autofluorescence measurements on thermal stressed and non-stressed corals using the same tissue homogenate dilution used to perform the present assay (see Supplementary Materials). Measurements were performed on a solid black 96-well plate using a multi-mode microplate reader (FilterMax F5, Molecular Devices). Results were normalized to the protein content of the sample, which was determined using a commercial reagent kit based on the Bradford assay (Sigma-Aldrich, ST. Louis, MO, USA). Results were expressed as peroxynitrite concentration (µM) / mg protein.

### 2.5. Data Presentation and Statistical Analyses

One-way ANOVAs were used to test: (i) if *Mu. harttii* and *Mi. alcicornis* shifted between feeding modes over time (PTMI data); (ii) if peroxynitrite levels significantly varied over time. If indicated, ANOVA was followed by the Student–Newman–Keuls multiple means procedure to detect statistically different (*p* ≤ 0.05) means. Data were checked for normality using Shapiro–Wilk’s test and for homoscedasticity using Levene’s test. Data were log transformed when necessary. To investigate a correlation between peroxynitrite and bleaching, and between the PTMI and sea surface temperature anomaly, a cross-correlation analysis for time series was performed. It was taken into account that data from time series tend to be autocorrelated, and that the degrees of freedom are overestimated, increasing Type I errors and the probability of detecting spurious associations when assuming a specified α value [34,35]. Thus, we corrected the number of degrees of freedom based on the sum of cross-products of the autocorrelations of tested variables over the examined lags [35,36], instead of removing the autocorrelation from the data. In the ‘Results’ section, Padj refers to P-value calculated using the corrected degrees of freedom. We only show the statistics for the lagged relationship with the highest correlation coefficient (R). To check if peroxynitrite levels and bleaching intensity were correlated for both species, we performed Pearson’s correlation, considering data acquired from the three reef sites and from the beginning of the monitoring period up to the onset of bleaching (higher than 40%). Peroxynitrite data were standardized among sites, equaling the highest level detected to one. Also, considering the many abiotic and biotic traits collected in the present study, and that principal component analysis (PCA) is an adequate method to integrate and interpret multiple data [37], we performed a PCA aiming to identify and summarize the correlation patterns among the multiple traits (bleaching, feeding plasticity, maximum temperature, degree heating weeks, sea surface temperature anomaly, and peroxynitrite). Also, in order to confirm the temporal patterns observed, the bleaching onset and recovery observed throughout the monitoring period was used as a discrete factor (‘before’, ‘during’, and ‘after’ bleaching) after scaling and centralizing the log-data using the prcomp function in vegan [38] package in the R environment (R Core Team 2013). The factoextra package [39] was used to visualize graphically the data through the function “fviz_pca_biplot”.

## 3. Results

### 3.1. Thermal Stress and Bleaching Frequency

**Figure 2:**
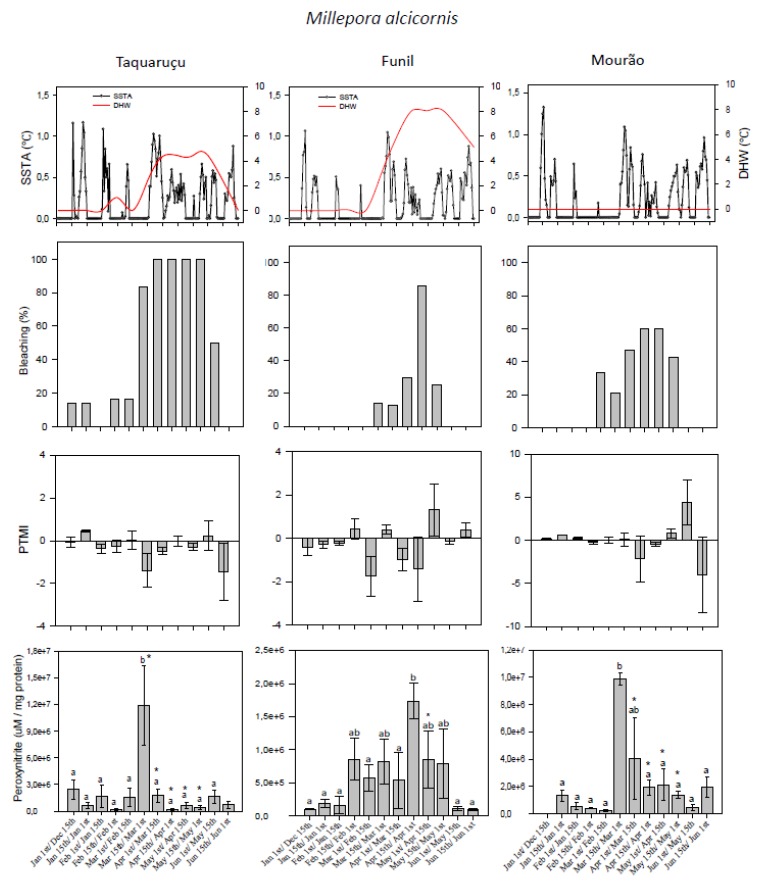
Thermal stress parameters [degree heating weeks (DHW) and daily sea surface temperature anomaly (SSTA)], bleaching frequency (%), Predominant Trophic Mode Index (PTMI), and peroxynitrite levels (µM/mg protein) for *Millepora alcicornis* at the three sites (Taquaruçu, Funil, and Mourão) of the Recife de Fora Marine Protected Area (Southwestern Atlantic), from January 2015 to June 2016, during El Niño Southern Oscillation. For peroxynitrite, different letters indicate significant differences over time (*p* ≤ 0.05), and asterisks (*) indicate more than 40% bleaching frequency observed. Data on PTMI and peroxynitrite are means ± s.e.m of three to four samples.

**Figure 3:**
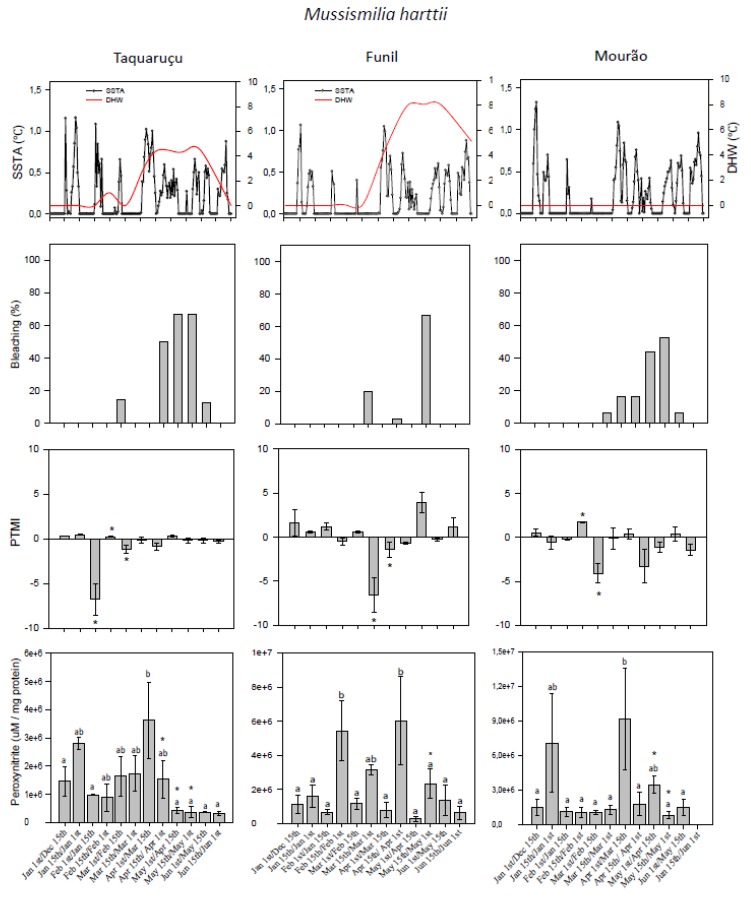
Thermal stress parameters [degree heating weeks (DHW) and daily sea surface temperature anomaly (SSTA)], bleaching frequency (%), Predominant Trophic Mode Index (PTMI), and peroxynitrite levels (µM/mg protein) for *Mussismilia harttii* at the three sites (Taquaruçu, Funil, and Mourão) of the Recife de Fora Marine Protected Area (Southwestern Atlantic), from January 2015 to June 2016, during El Niño Southern Oscillation. For PTMI data, positive and negative values indicate prevalence of autotrophy and heterotrophy, respectively, with asterisks (*) indicating significant shifts of feeding mode prevalence over time (*p* ≤ 0.05). For peroxynitrite data, different letters indicate significant differences over time (*p* ≤ 0.05) and asterisks (*) indicate more than 40% bleaching frequency observed. Data on PTMI and peroxynitrite are means ± s.e.m of three to four samples.

Seawater temperature measured during the monitoring period for each of the sites is summarized in Table 1. Sea surface temperature anomaly (SSTA) was observed throughout the monitoring period for all sites (Figure 2 and Figure 3). SSTA positive deviations ranged from 0.1 to 1.1 °C at Taquaruçu, from 0.1 to 1.0 °C at Funil, and from 0.1 to 1.3 °C at Mourão. 

Critical thermal stress for corals, indicated by degree heating weeks (DHW) values of 4.5 °C-weeks, was observed from April to May at Taquaruçu site (Figure 2 and Figure 3). For this locality, *Mi. alcicornis* showed signs of bleaching during the entire monitoring period, except for the second half of January and first half of June, while 100% bleaching was observed from mid-March to mid-May (Figure 2). *Mussismilia harttii* showed bleaching signs mainly from April to May, with highest bleaching frequency (67%) from mid-April to May (Figure 2).

DHW values of 6 °C-weeks or greater were observed from April to mid-June at Funil site (Figure 2 and Figure 3). Bleaching was observed for *Mi. alcicornis* from March to May, with the highest bleaching frequency (86%) observed in mid-April. In turn, *Mu. harttii* showed bleaching signs between March (20%) and mid-May (67%) (Figure 3). 

No critical thermal stress indicated as DHW was observed for the site Mourão (Figure 2 and Figure 3), however, maximum temperatures (up to 34.2 °C) were registered for this location. Bleaching for *Mi. alcicornis* was observed from mid-February to May, with the highest bleaching frequency (60%) in May. *Mu. harttii* bleached from March to May, with the highest bleaching frequency (53%) observed in the first half of May (Figure 3).

### 3.2. Predominant Trophic Mode Index 

Significant shifts of PTMI were observed for *Mu. harttii* at all sites (ANOVA, 1.19 ≤ F ≤ 1.631, 0.001 < *p* ≤ 0.006, Figure 3). At Taquaruçu site, a significant predominance of heterotrophy was observed during the second half of January and February, while autotrophy was predominant during the first half of February (SNK, *p* < 0.001). At Funil, heterotrophy was significantly dominant during the entire month of March (SNK, *p* < 0.001), while autotrophy prevailed the rest of the monitoring period. At Mourão site, autotrophy prevailed during the first half of February (SNK, *p* = 0.05). However, it was followed by a significant heterotrophy peak in the second half of February (SNK, *p* = 0.006). Cross-correlation analysis shows that SSTA was associated with PTMI for *Mu. harttii* for all three sites (−0.783 ≤ R ≤ −0.638, 0.004 ≤ Padj ≤ 0.05, 9.4 ≤ df ≤ 10.8) with a lag of 15 days for Taquaruçu and Funil, and of 30 days for Mourão, which means that a peak in sea surface temperature anomaly preceded the heterotrophy peak by 15 or 30 days (Table 2, Figure 3). On the contrary to *Mu. harttii, Mi. alcicornis* displayed a near-equilibrium between feeding modes at all sites, with PTMI values not varying significantly (ANOVA, 3.62 ≤ F ≤ 12.38, 0.16 ≤ *p* ≤ 0.34, Figure 2). Accordingly, no correlation between PTMI and SSTA (*P* ≥ 0.05) was observed for all collecting sites (Table 2). 

### 3.3. Peroxynitrite (ONOO^−^) Generation

Significant differences in ONOO^−^ levels over time were observed for *Mi. alcicornis* (ANOVA, 3.25 ≤ F ≤ 4.555, 0.001 < *p* ≤ 0.007, Figure 2) and *Mu. harttii* (ANOVA, 2.235 ≤ F ≤ 5.121, 0.001 < *p* ≤ 0.045, Figure 3) at all sites. *Mi. alcicornis* showed a significant increase in ONOO^-^ production in the first half of May at Taquaruçu (SNK, *p* ≤ 0.004) and Mourão (SNK, *p* ≤ 0.003), and in the first half of April at Funil (SNK, *p* ≤ 0.017). *Mu. harttii* presented significantly higher levels of ONOO^-^ in the second half of March at Taquaruçu (SNK, *p* ≤ 0.044) and in first half of May for Mourão (SNK, *p* ≤ 0.043). Two peaks of ONOO^-^ production at Funil were observed in the first half of February and March (SNK, *p* ≤ 0.041). In all cases, ONOO^−^ generation significantly decreased after bleaching peaks (Figure 2 and Figure 3).

**Figure 4:**
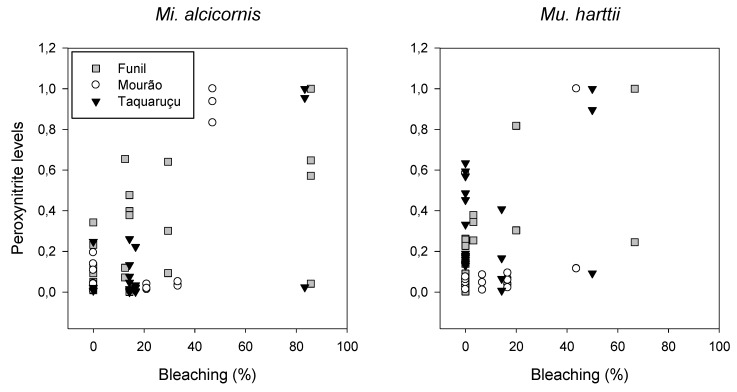
Correlation plot of peroxynitrite levels (standardized data) versus bleaching frequency for *Millepora alcicornis* and *Mussismilia harttii,* considering data from the three sites together. Highest peroxynitrite and bleaching levels were adjusted according the time lag observed for each of the sites, which varied from 0 to 30 days. A significant correlation was observed between ONOO^−^ levels and bleaching intensity for *Mi. alcicornis* (*p* < 0.001, Pearson correlation coefficient = 0.81) and *Mu. harttii* (*p* = 0.001, Pearson correlation coefficient = 0.64).

A significant correlation was observed between ONOO^−^ levels and bleaching intensity for *Mi. alcicornis* (*p* < 0.001, Pearson correlation coefficient = 0.81) and *Mu. harttii* (*p* = 0.001, Pearson correlation coefficient = 0.64) (Figure 4). 

Cross-correlation analysis indicated species-specific time lags between peroxynitrite generation and bleaching for Taquaruçu and Funil. Bleaching and peroxynitrite were correlated with no lag (R = 0.808, Padj = 0.003) and a 15 days lag (R = 0.63, Padj = 0.04) for *M. alcicornis* from the locality of Funil (df = 7.3) (Table 2). Peroxynitrite and bleaching were also correlated in *M. harttii* with a 15 days lag both at Funil (R = 0.66, Padj = 0.03,) and Taquaruçu (R = 0.75, Padj = 0.01, df = 6.8; Table 2). No correlation between peroxynitrite generation and bleaching was observed in Mourão (P > 0.05), where no cumulative thermal stress (as DHW) was detected, and a lower bleaching frequency was observed for both species. 

### 3.4. Principal Component Analysis 

**Figure 5:**
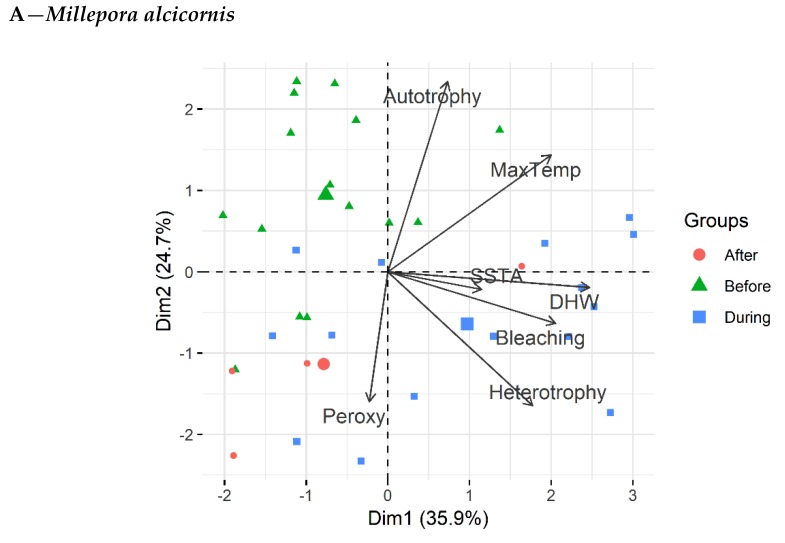

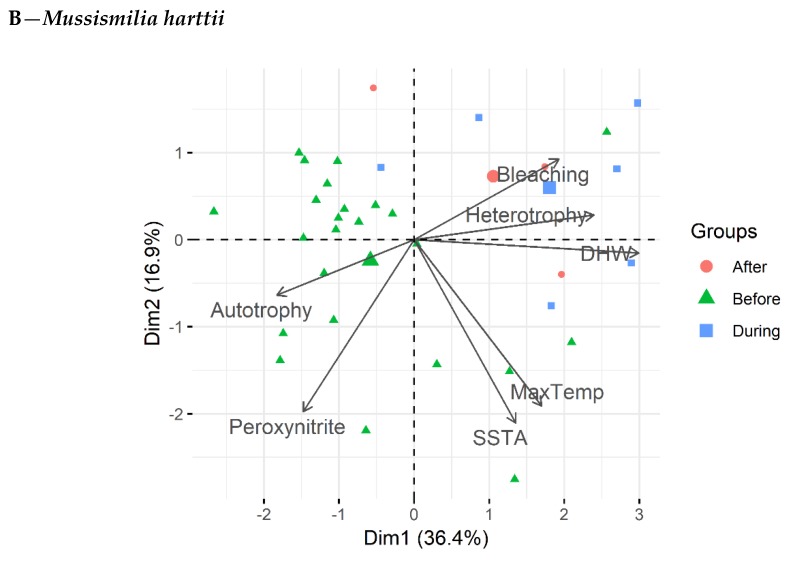
Principal component analysis of bleaching, degree heating weeks (DHW), sea surface temperature anomaly (SSTA), maximum temperature (MaxTemp), autotrophy, heterotrophy, and peroxynitrite in *Millepora alcicornis* (**A**) and *Mussismilia harttii* (**B**). All traits were grouped into 3 clusters: after, before, and during the bleaching event. The two main eigenvectors retained, respectively, 60.6% (Dim1 = 35.9%; Dim2 = 24.7%) and 53.3% (Dim1 = 36.4%; Dim2 = 16.9%) of the total variance.

All traits were grouped together [bleaching, maximum temperature (MaxTemp), degree heating weeks (DHW), sea surface temperature anomaly (SSTA), peroxynitrite, auto- and heterotrophy] into a multivariate analysis (PCA) for *Mi. alcicornis* and *Mu. harttii*. The two main eigenvectors retained, respectively, 60.6% (Dim1 = 35.9%; Dim2 = 24.7%) and 53.3% (Dim1 = 36.4%; Dim2 = 16.9%) of the total variance (Figure 5A,B). Bleaching, heterotrophy, and DHW were positively correlated with Dim1 (0.60 < R < 0.90) for both species. In turn, while SSTA was strongly correlated with DHW for *Mi. alcicornis* (Dim1, 0.41 < R < 0.89), it was more associated with MaxTemp for *Mu. harttii* (Dim1, 0.40 < R < 0.51). Peroxynitrite was associated with autotrophy for *Mu. harttii* into Dim1 (−0.50 < R < −0.54)*,* while it was correlated with heterotrophy for *Mi. alcicornis* into Dim2 (−0.57 < R < −0.59). When bleaching is used as a discrete factor (‘before’, ‘during’, and ‘after’), autotrophy was grouped ‘before’ the bleaching event for *Mu. harttii*, and ‘before’ and ‘during’ for *Mi. alcicornis*. Heterotrophy, however, was grouped during the bleaching event for both species. In turn*,* peroxynitrite was grouped ‘before’ the bleaching event for *Mu. harttii*, and ‘before’ and ‘during’ for *Mi. alcicornis*, which goes in agreement with the different time lags observed for each species.

## 4. Discussion

This study brings new insights into the mechanisms involved in the breakdown of the cnidarian–dinoflagellate symbiosis during a natural bleaching event under El Niño influence. We showed that high peroxynitrite (ONOO^−^) levels are associated with bleaching occurrence and intensity, with however a species-specific time lag. Also, this study brings the first evidence of a thermally induced shift from auto- to heterotrophy in the scleractinian coral *Mu. hartii*, while the hydrocoral *Mi. alcicornis* was unable to show such nutritional plasticity. 

### 4.1. Thermal Stress, Bleaching Frequency, and Feeding Mode Plasticity

A positive correlation between large-scale bleaching (>50%–60% of the colonies investigated) and DHW was observed for both coral species in two out of the three monitored sites (Taquaruçu and Funil), in agreement with previous observations [2,29]. The third site (Mourão), which didn’t experience DHW, presented a lower bleaching intensity (<60% of the colonies investigated), although still half of the coral colonies bleached during the highest thermal anomalies recorded. Such lack of correlation between bleaching and DHW was previously observed [40], and was related to the occurrence of small increases in temperature (lower than 1 °C above monthly mean) persisting over months. The comparison of the seawater temperature profiles of the three sites, however, suggests that bleaching at Mourão may be related to the highest sea surface temperature anomaly (SSTA) recorded among sites (+1.3 °C) and to other environmental stressors such as water quality. Mourão is indeed located in the most landward side of Recife de Fora conservation reef area, showing twofold higher average levels of nitrogen (up to 1.16 µmol L^−1^) than the seaward side—Taquaruçu [41]. Due to the fact that high levels of nitrogen in seawater have been linked to reduction in the bleaching threshold [33], high nitrogen concentration at Mourão could explain the bleaching occurrence, in the absence of DHW. 

Bleaching frequency was also species-specific, with *Mi. alcicornis* displaying twice higher bleaching frequency (up to 100%) at all sites compared to *Mu. harttii*. In agreement with our observation, hydrocorals were among the first reef-building species to undergo bleaching during the El Niño event (1982–1983) of the Pacific coast of Panama [42]. In addition, a recent study on South Atlantic Ocean reefs reported *Millepora* spp. as the most affected species during the bleaching events of 2016 and 2017 [43]. The higher sensitivity of hydrocorals to thermal stress compared to some scleractinian coral species, as observed here and in the aforementioned studies, may be related to their lower heterotrophic capacity. While the scleractinian coral *Mu. harttii* significantly shifted between feeding modes, *Mi. alcicornis* trophic mode remained unaffected during the entire monitoring period. Previous studies have reported that corals rely on heterotrophy as a compensation mechanism for the reduced autotrophy resulting from bleaching [8,9,10,44]. As the size of corallites has already been described as a proxy for heterotrophic capacity [45], the high degree of heterotrophy in *Mu. harttii* may be explained by its large corallite, with a diameter of 15 mm or larger [46], coupled with large tentacles, which also favor predation [47]. This is 50-fold greater than the gastropore diameter found in *Mi. alcicornis* (0.3 mm) [48]. Therefore, *Mu. harttii* may have more resources than *Mi. alcicornis* for enduring thermal stress events. The shifts to heterotrophy in *Mu. harttii* were indeed correlated with the occurrence of sea surface temperature anomalies (SSTA). These findings are in concert with previous reports for other scleractinian coral species [10,49,50] and can be associated with variability in oceanographical and meteorological phenomena [10,51,52]. 

### 4.2. Peroxynitrite Generation and Bleaching

It has been hypothesized that the deleterious effects of high levels of nitric oxide (NO) during bleaching are related to its conversion to ONOO^−^, which acts as a cytotoxic compound. Specifically, ONOO^−^, through mitochondrial membrane damage, may be responsible for the release of potent proapoptotic molecules that initiate an apoptotic cascade involved in the bleaching process [14,15] In vivo production of ONOO^−^ in the symbiotic sea anemone *Exaiptasia pulchella* indeed occurred under thermal stress; nonetheless, owing to the nonsignificant effect of ONOO^−^ scavenger, its generation was considered insufficient to influence symbiont loss [16]. In contrast, our results bring the first evidence that ONOO^−^ is an important mediator of bleaching in scleractinian corals and hydrocorals experiencing heat stress under field conditions. High levels of ONOO^−^ were indeed detected shortly before the maximum bleaching frequency for both species at all sites (Figure 2 and Figure 3) and a significant correlation was observed between the highest levels of ONOO^−^ and the highest bleaching frequency (Figure 4). Additionally, species-specific time lag responses were detected through cross-correlation analysis regarding maximum ONOO^−^ generation and maximum bleaching frequency, from 0 to 30 days prior bleaching. The different time lags found for each species, greater for *Mu. harttii* (up to 30 days before) and shorter for *Mi. alcicornis* (up to 15 days before), were also reinforced by PCA that correlated ONOO^−^ before bleaching occurrence for *Mu. harttii* and during bleaching occurrence for *Mi. alcicornis*.

The species-specific time lag response for ONOO^−^ generation and bleaching occurrence in the present study suggests a differential susceptibility to ONOO^−^ and/or capacity to neutralize and counteract the deleterious effects of this highly reactive RNS. For example, the antioxidant capacity has been demonstrated as a critical component of stress tolerance in scleractinian corals [53] and antioxidant capacity has been reported to be approximately threefold lower in *Mi. alcicornis* than in *Mu. harttii* [54]. Therefore, the lower antioxidant capacity observed in *Mi. alcicornis* can explain why this species present a smaller interval between ONOO^−^ generation and bleaching, while *Mu. harttii*, with a higher antioxidant capacity, presents a repeated and longer interval between ONOO^−^ and bleaching. In addition, these species-specific time lags and antioxidant capacities are aligned with the different bleaching frequencies observed (higher and lower for *Mi. alcicornis* and *Mu. harttii*, respectively).

Also, it is worth noting that most studies on the role of RNS in the bleaching mechanisms were performed with laboratory experiments, which could differ from the present study with corals undergoing bleaching in the field [16]. Finally, despite the fact that the present study cannot prove that alternative bleaching pathways directly involving ROS or NO are as critical as ONOO^−^, we propose that this highly cytotoxic molecule may exert a more relevant influence in cnidarians’ thermal bleaching process than previously suggested. 

## 5. Conclusions

Our findings show that ONOO^−^ may be an important mediator of bleaching in scleractinian corals and hydrocorals experiencing heat stress under field conditions, with consistent observation of higher levels preceding maximum bleaching occurrence in a species-specific time lag. Measuring ONOO^−^ may be an additional tool to quantitatively assess the stress level in corals, similar to chlorophyll and ROS. Further investigations are, however, needed to bring detailed insights into the mechanisms of ONOO^−^ involved in the onset of bleaching. In addition, we show that SSTA may increase heterotrophic feeding and that the higher sensitivity of hydrocorals to thermal stress may be related to their lower heterotrophic input. The overall results may further the understanding of the biochemical mechanisms associated with bleaching, as well as of the trophic behavioral response that cnidarians may display when under thermal stress.

## Figures and Tables

**Table 1:** Seawater surface temperature (°C) during the 6 month (from January 2015 to June 2016) monitoring period in the three sites of the Recife de Fora Marine Protected Area (Southwestern Atlantic) during the El Niño Southern Oscillation.

Reef Site	Taquaruçu	Funil	Mourão
Minimum temperature	20.5	22.9	22.8
Maximum temperature	27.5	31.1	34.2
Median	24.7	26.5	26.7
Mean	24.7	26.5	26.7
Standard deviation	1.0	1.0	0.9
Standard error	0.2	0.2	0.2

**Table 2:** Cross-correlation analysis testing i) the relationship between sea surface temperature anomaly and trophic mode, and ii) peroxynitrite and bleaching. P-values (P_adj_) were calculated based on adjusted degrees of freedom (df_adj_) accounting for the autocorrelation of time series. Statistical descriptors are shown for the lagged relationship with the highest correlation coefficient (R). -, Correlation coefficient not significant at any lag. Bold numbers indicate significant *p* values. * indicate marginally significant *p* values.

**Site**	**Sea surface temperature anomaly *x* trophic mode**							
	*Millepora alcicornis*				*Mussimillia harttii*			
	Lag (days)	R	P_adj_	df_adj_	Lag (days)	R	P_adj_	df_adj_
Taquaruçu	-	−0.218	0.53	10.2	15	−0.692	**0.03**	9.5
Funil	-	−0.401	0.2	11.1	15	−0.638	**0.05**	10.8
Mourão	-	−0.577	0.053	11.4	30	−0.783	**0.004**	9.4
	**Peroxynitrite *x* bleaching**							
Taquaruçu	-	−0.27	0.4	8.6	15	0.75	**0.01**	6.8
Funil	0 / 15	0.63 / 0.81	**0.003/0.04**	7.3	15/30	0.66 / 0.62	**0.03** / 0.06 *	7.6
Mourão	-	0.36	0.39	8.2	-	0.09	0.79	8.5

## Supplementary Materials

The following are available online at https://www.mdpi.com/2076-2607/7/10/426/s1, Table S1: Autofluorescence data detected at Ex/Em = 490/530nm (fluorescence/mg protein, mean ± SEM, N = 6) of two coral species, the scleractinian *Mussismilia harttii* and the hydrocoral *Millepora alcicornis*, before (samples collected from December to March) and during thermal stress (April to June), and their statistical descriptors. No significant differences in fluorescence between thermal stressed and non-stressed corals, for each species, were indicated by Student’s t-test.
