# Supplementary material for: Peroxynitrite Generation and Increased Heterotrophic Capacity Are Linked to the Disruption of the Coral–Dinoflagellate Symbiosis in a Scleractinian and Hydrocoral Species
Source: Microorganisms. 2019 Oct 9;7(10):426. doi: 10.3390/microorganisms7100426 (PMC6843776; doi:10.3390/microorganisms7100426)
Supplement: Supplementary file 1 [file microorganisms-07-00426-s001.pdf]

Supplementary material

Table 1. Autofluorescence data detected at Ex/Em = 490/530nm (fluorescence/mg protein, mean ± SEM, N = 6) of two coral species, the scleractinian *Mussismilia harttii* and the hydrocoral *Millepora alcicornis,* before (samples collected from December to March) and during thermal stress (April to June), and their statistical descriptors. No significant differences in fluorescence between thermal stressed and non-stressed corals, for each species, were indicated by Student's t-test.

	**Condition**		**Statistical descriptors**		
**Species**	Before thermal stress	During thermal stress	df	t	*p*-value
Mi. alcicornis	4172.9 ± 835.5	3511.8 ± 595.2	10	0.64	0.534
Mu. harttii	20522.1 ± 3347.8	20963.4 ± 6552.8	10	-0.06	0.953
